# Midterm Results of Thoracic Endovascular Aortic Repair with Reentry Closure for Chronic Type B Aortic Dissection with Aneurysmal Dilatation

**DOI:** 10.3400/avd.oa.22-00065

**Published:** 2022-12-25

**Authors:** Kiyoshi Chiba, Hiroshi Nishimaki, Yukihisa Ogawa, Masahiro Tomita, Ryuji Nakamura, Satoshi Kinebuchi, Shota Kita, Masahide Komagamine, Kan Nawata, Masahide Chikada, Takeshi Miyairi

**Affiliations:** 1 Department of Cardiovascular Surgery, St Marianna University School of Medicine, Kawasaki, Kanagawa, Japan; 2 Department of Radiology, St Marianna University School of Medicine, Kawasaki, Kanagawa, Japan

**Keywords:** aortic dissection, thoracic endovascular aortic repair, reentry closure, false lumen thrombosis

## Abstract

**Objectives:** This study aims to discuss the midterm results of thoracic endovascular aortic repair (TEVAR) with reentry closure for chronic type B aortic dissection (CTBAD).

**Materials and Methods:** This retrospective study analyzed 13 patients with CTBAD who underwent TEVAR with reentry closure between July 2014 and December 2020. We evaluated the false lumen (FL) cross-sectional area using computed tomography images of the descending aorta at the level of the bronchial bifurcation, Valsalva sinus, celiac artery, and infrarenal abdominal aorta pre- and postoperation. The study endpoints were technical and clinical success rates, freedom from additional aortic reintervention or surgery, and survival.

**Results:** Technical success was obtained in 12 patients (92.3%) with no hospital mortality and neurological complications. The postoperative observation period was 49.2±21.5 months. The clinical success rate was 76.9% (10 cases), and a postoperative reduction of the FL cross-sectional area was obtained in 53.8% of patients. The 5-year overall survival rate was 64.8% with no aortic-related deaths while the 5-year freedom from additional aortic surgery rate was 66.7%.

**Conclusions:** TEVAR with reentry closure suggests preventing FL dilatation or rupture in CTBAD, but the revision of our devices and further research with more patients and longer follow-up periods are required.

## Introduction

Persistent pressurization of the false lumen (FL) leads to late aortic expansion in 35% of the patients undergoing thoracic endovascular aortic repair (TEVAR) for chronic type B aortic dissection (CTBAD).^1)^ In addition, the cumulative rupture rate is 30%, and the 5-year survival rate is 50%–80% when the aortic aneurysm hits 60 mm in size.^2)^

For complicated (i.e., involving aortic enlargement and rupture) CTBAD, TEVAR is the first treatment of choice according to the 2014 European Society of Cardiology guidelines.^3)^ Even if the primary entry is closed, the FL pressurization from persistent retrograde flow to the intercostal artery and abdominal branches from reentry can lead to late aortic events, including rupture, in 35% of the patients undergoing TEVAR for CTBAD.^4)^ Complete FL thrombosis has been reported as a prophylactic factor for the rate of aortic event occurrence.^5)^

Earlier on, we described the initial results of TEVAR, which uses the Candy-Plug method in combination, to control the blood flow of the blowup from the reentry.^6,7)^ We considered the midterm results of the TEVAR with reentry closure for CTBAD to attain complete FL thrombosis in the latest study.

## Materials and Methods

### Study design

The institutional review board of St. Marianna University School of Medicine (approval number 5679) approved this retrospective study. This study comprised 228 patients with CTBAD who underwent initial TEVAR at our institution. Among these, we evaluated 13 patients that include 9 patients with residual dissection after prior type A repair with patent FL (double barrel)-type aortic dissection who underwent TEVAR with reentry closure from July 2014 to December 2020. The indications were rupture complication (n=1) and uncomplicated cases with either aortic diameter >55 mm or rapid expansion (5 mm/6 months).

In this cohort, three patients with ascending aorta replacement and six with total arch replacement, who did not undergo the frozen elephant trunk technique, were included in type A aortic dissection. In type B aortic dissection, four patients who had completed more than 1 year from onset without preemptive TEVAR were included.^8)^

Out of the six cases in the single-stage operations, in which primary and reentry closure operations were performed simultaneously, four cases were of type A aortic dissection after surgery and two were of type B aortic dissection following optimal management treatment for more than one year since the initial dissection. Of seven cases in the two-staged operations, two required descending aorta replacement, and five required isolated TEVAR as the primary entry closure and an additional treatment as reentry closure for FL expansion, which resulted from persistent reentry flow from the abdominal branch.

### Study endpoint

We gauged technical success (accurate deployment) and clinical success (complete FL thrombosis on follow-up computed tomography [CT]), complications, change in the FL cross-sectional area, survival rate, and freedom from additional intervention. The FL diameter and area were measured on pre- and postoperative CT angiographic images at the levels of the bronchial bifurcation, Valsalva sinus, celiac trunk, and infrarenal abdominal aorta in the analysis of aortic morphological change. Aortic remodeling was defined as at least a 5% reduction in the FL cross-sectional area in the CT images relative to the latest measurement before the reentry closure.^9)^ The diameters of oval- or crescent-shaped FLs were calculated by averaging the long and short-axis diameters using Osirix software. The cross-sectional area was calculated as the product of its radius and π. Additional midterm endpoints included survival rate and freedom from additional aortic intervention (post-reentry closure). The postoperative observation period was defined as the time from reentry closure to the latest CT.

### Surgical procedure

All procedures were carried out in a hybrid operating room with a fixed imaging system and under general anesthesia. The size of the proximal site was selected to be an excess of 10%–15% of the maximum short axis without dissection.

The size of the distal site was selected to be an excess of 5% or the same size as the long axis of the true lumen site. Valiant Closed Web (Medtronic Vascular, Santa Rosa, CA, USA) was placed in a straight position at the distal site, and a comfortable TAG® (CTAG) (W. L. Gore & Associates, Flagstaff, AZ, USA) was placed at the proximal site. Then, we used it in the proximal and distal sites after obtaining active control CTAG.

We evaluated the true lumen and FL as well as the position of entry and origin of abdominal branches using intravascular ultrasonography. We performed the reentry closure with the Candy-Plug (n=10), coil embolization (n=4), and spot stenting for reentry sites of abdominal branches (n=4) and endovascular aortic repair including the Double D technique for infrarenal abdominal arteries and iliac arteries (n=5), considering the existence of a reentry approaching the FL on preoperative contrast CT.^10)^ The details of reentry closure are shown in **Table 1B** and **Fig. 1A**.

**Table 1A:** Table 1A Patients’ demographics and concomitant morbidities for open surgery

Number of patients	13
Interval	Jul, 2014–Dec, 2020
Men (%)	10 (76.9)
Age (years)	65.2±9.7 (51–79)
Aneurysm diameter (mm)	57.5±6.8 (44–68)
Residual type A/Type B	9/4
Respiration dysfunction [forced expiratory volume in 1 second (FEV1.0)<1.2 L] 【Including home oxygen therapy】	7【3】
History of left thoracotomy	3
Post tracheotomy	1
Steroid used	1

**Table 1B:** Table 1B Summary of reentry closure

Single-stage operations				
Age (years)	Surgical history	Primary entry close	Origin of reentry	Reentry closure (refer to **Fig. 1A**)
66	none	TEVAR	Lt. RA, LA	(a) (CP)
59	Y graft	TEVAR	Celiac, ICA, Rt, RA	(b), (f) (CP, Coil for celiac, Spot stenting for SMA, Petticoat using AFX cuff)
74	AAR	TEVAR	Celiac, ICA, LA	(i) (Coil for celiac, Ex-cuff for ICA, Petticoat using AFX cuff)
67	TAR	TEVAR	ICA, LA, IMA	(j) (Coil for ICA and IMA, Ex-cuff for ICA, EVAR for LA)
55	Bentall+TAR	TEVAR	ICA, LA, Rt, RA,	(a) (CP)
77	AAR	TEVAR	ICA, LA, Rt, RA, Lt EIA	(a) (CP)
Two-staged operations				
Age	Surgical history	Primary entry close	Origin of reentry	Reentry closure (refer to **Fig. 2A**)
59	none	DAR	Anastomosis, Celiac trunk Iliac artery	(c) (SITCIF, EVAR)
78	AAR, TAR	TEVAR	Celiac, Rt. RA, LA	(k) (Coil for ICA, IMA, CP, Spot stenting, EVAR)
73	TAR, DAR	TEVAR	Anastomosis, ICA, Lt. RA, LA	(b) (g) (CP, Ex-cuff for ICA, Petticoat using AFX cuff)
51	AAR	TEVAR	Rt RA, IMA, LA, Rt. iliac	(h) (CP, Petticoat using AFX cuff, EVAR)
60	Bentall+MVR TAR	TEVAR	Lt. RA, LA, iliac	(a) (CP)
52	Bentall, TAR	TEVAR	Celiac, Lt. RA	(a) (CP)
79	none	TEVAR	LA, IMA, iliac	(l) (CP, EVAR)

AAR: ascending aorta replacement; TAR: total arch replacement; DAR: descending aorta replacement; MVR: mitral valve replacement; ICA: intercostal artery; LA: lumber artery; SMA: superior mesenteric artery; RA: renal artery; SITCIF: stenting into true lumen coiling into false lumen; CP: Candy-Plug; Ex-cuff: Excluder aortic cuff; EVAR: endovascular aortic repair; IMA: inferior mesenteric artery; Rt.: right; Lt.: left; TEVAR: thoracic endovascular aortic repair; EIA: external iliac artery

**Figure 1A:**
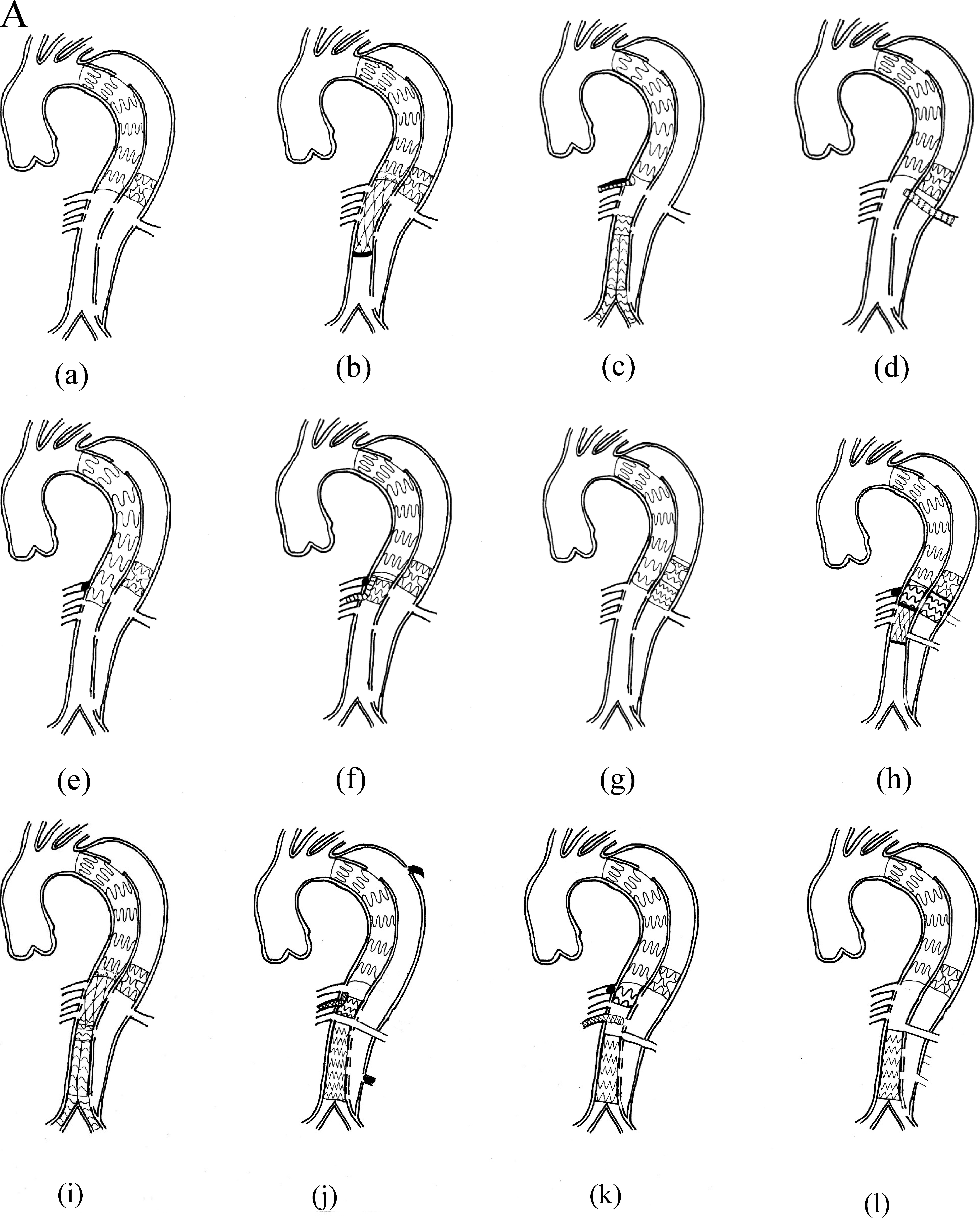
Fig. 1A Details of the reentry closure. (**a**) A simple Candy-Plug method is used in seven cases. (**b**) When the Candy-Plug is detained in the false lumen (FL) at the abdominal branch level in the true lumen. Petticoat technique with modified AFX is used in combination in three cases. Procedure of reentry closure in the abdominal branch: (**c**), (**d**), (**e**), (**f**). When the branched arteries were complete perfusions of the FL or both the FL and true lumen, we confirmed the anatomical traffic through the gastric duodenal artery between the celiac trunk and superior mesenteric artery. Spot stenting method, chimney method, and the coil embolization of the FL were performed in combination. The renal artery was used by spot stenting method. (**g**), (**h**) The intercostal artery is closed by using Excluder aortic cuff at FL side. Procedure of reentry closure in infra renal abdominal aorta: (**i**), (**j**), (**k**), (**l**). The reentry, such as lower mesenteric artery and lumber arteries, are closed using endovascular aortic repair including Double D technique. Concretely, the Endurant aortic extension (Medtronic Vascular, Santa Rosa, CA, USA) is placed from under the renal artery to the terminal abdominal aorta or the common iliac artery.

**Figure 1B:**
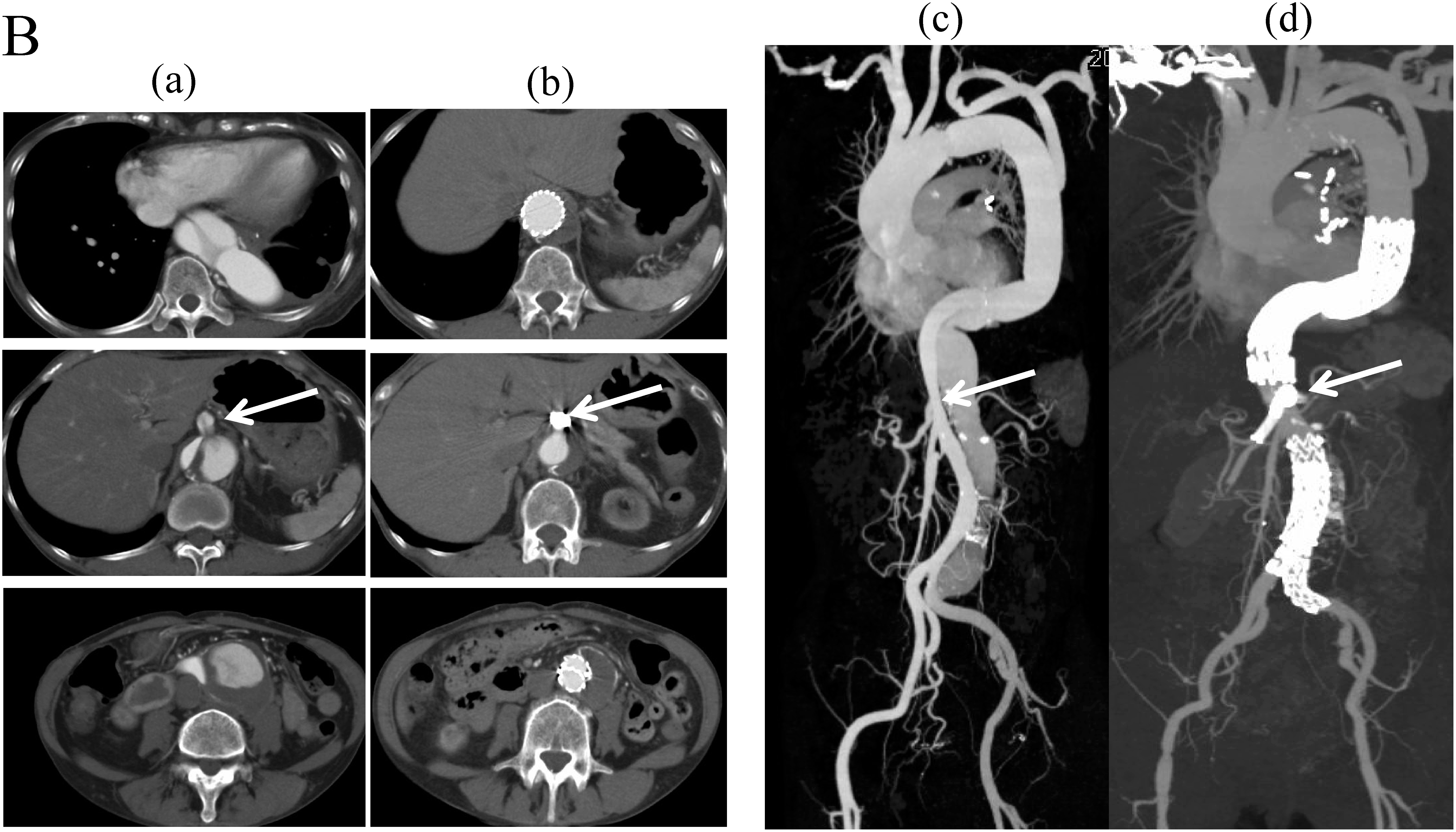
Fig. 1B A woman in her 50s after being treated with descending aorta replacement after chronic type B aortic dissection. (**a**), (**c**) Preoperative contrast enhanced computed tomography (CECT; axial and 3-dimensional) shows reentry points in the distal anastomosis, celiac trunk, lumber artery, left common iliac artery. (**b**), (**d**) CECT three years after thoracic endovascular aortic repair with reentry closure shows complete false lumen thrombosis and the reduction of false lumen area.

The details of reentry closure are as follows:

①Candy-Plug method (**Fig. 1A-a**): We selected the size of the Excluder Aortic cuff (W. L. Gore & Associates, Flagstaff, AZ, USA) to be 10%–15% greater than the average of the diameters of the long and short axes in the FL.^11,12)^ The Candy-Plug technique, which occludes the FL, is a method of achieving FL thrombosis by controlling the FL’s blood flow from reentry. The prerequisite preoperative diagnostic imaging conditions for using the Excluder include reentry below the diaphragm with an approach to the FL, and the mean FL diameter should be ≤34 mm.

We preferred deploying stent-grafts or bare stents at the same level as the true lumen to prevent suppression of the true lumen if the Candy-Plug gets detained in the FL at the abdominal branch level (**Fig. 1A-b**). If a minor entry was present due to multiple intercostal arterial reentries to the celiac trunk, stent-grafts were placed in the true lumen from the proximal side to the celiac trunk level. The size of the stent-graft was approximately the same as that of the long axis of the true lumen at the deployment site.

②Approach for abdominal branch reentry (**Figs. 1A-c–1A-f**): This procedure’s main purpose is to control the FL’s persistent blood flow by pulling out the abdominal branches without blocking the blood flow in the true lumen. We detained a bare stent the size of the maximum long axis of the branch part^13)^ that coils for FL throughout the space of the bare stent when the branched arteries were perfusions of both the true lumen and FL (**Figs. 1A-c** and **1B**). A covered stent was not used in this approach. We named this method stenting into the true lumen and coiling into the FL (SITCIF).^14)^ In cases of the renal arteries, a bare stent was only placed in the true lumen if it was narrowed by a static lesion in preoperative CT (**Fig. 1A-d**).

In cases where the branched arteries were complete perfusions of the FL, the following procedure was performed: The celiac trunk was obstructed by the stent-graft if the blood flow from the superior mesenteric artery to the celiac trunk via the gastric duodenal artery was confirmed by preoperative three-dimensional CT and intraoperative distal subtraction angiography. Superior mesenteric and renal arteries were excluded from this procedure. In addition, coil embolization for the origin of the celiac trunk was performed throughout the middle colic artery via the superior mesenteric artery to prevent type 2 endoleaks (**Figs. 1A-e, 1A-f, 1A-h, 1A-k**).^15)^

Furthermore, we performed the following procedure when the intercostal artery was withdrawn between the abdominal branched arteries: We occluded the origin of the intercostal artery using an Excluder aortic cuff while saving branched vessels by the chimney technique. This procedure requires an adequate anatomical distance between the abdominal branches and the intercostal artery (**Fig. 1A-f**).^15–17)^ If there was a concern about the branched blood vessel being closed by this procedure, Excluder aortic cuff was detained on the FL side to close the origin of the intercostal artery (**Figs. 1A-g** and **1A-h**).

③Reentry closure for infrarenal abdominal artery (**Figs. 1A-c, 1A-i, 1A-j, 1A-k, 1A-l**, and **1B**): The true lumen of this site is narrowed by an enlarged FL. In such cases, we performed the reentry closure using the Double D technique. Concretely, the Endurant aortic extension (Medtronic Vascular, Santa Rosa, CA, USA) is placed from under the renal artery to the terminal abdominal aorta. Then, the Endurant aortic extension is placed just before the origin of the bilateral internal iliac artery when necessary.^18)^

### Statistical analysis

Data comparisons were performed using paired t-tests. P-values<0.05 were considered statistically significant. The survival rate and the freedom from additional aortic reintervention were analyzed using the Kaplan–Meier method.

## Results

The average age of the participants was 65.2±9.7 (mean±standard deviation) years, which ranges from 51 to 79 years, and the average sac diameter was 57.5 mm±6.8 mm, which ranges from 52 to 68 mm. There were seven patients with reduced respiratory function (forced expiratory volume in 1 second [FEV 1.0]<1.2 L) and 3 patients with a history of left thoracotomy because of lung surgery (**Table 1A**).

The clinical results of this study are shown in **Table 2A**. The technical success rate was 92.3%. In one case, the Excluder was deployed diagonally on the long axis of the FL. None of the patients died within 30 days in the hospital or had neurologic complications, vascular injury, or respiratory dysfunction. The period from onset to the single-stage operations was 108.5±75.2 months, which ranges from 12.6 to 174.5 months. The period from onset to primary entry closure was 24.1±20.4 months (range: 7.2–57.2 months) and from primary entry closure to reentry closure was 40.7±35.1 months (range: 9.1–97.2 months) in the two-staged operations. The average observation period was 49.2±21.5 months (range: 19–66 months) in all cases. Additional TEVAR or additional surgery was performed in four cases during a follow-up after >1 month. Additional TEVAR was needed two times in one case. The first additional TEVAR was performed on a postoperative day (POD) 399 because of a new entry tear at the distal site. And second additional TEVAR was performed on POD 1225 because of a type 3b endoleak. In addition, additional treatment was required for endoleaks caused by the Candy-Plug in two cases. In one case, we placed an additional Candy-Plug because of the type 1b endoleak around the first Candy-Plug, between the Ex-cuff and the external membrane, on POD 529. In the other case, the FL diameter expanded due to a type 1b endoleak. Thus, we performed additional aortic surgery to replace the descending aorta and removed the Candy-Plug on POD 486. The last patient required total arch replacement because of retrograde type A aortic dissection on POD 2023 despite achieving complete FL thrombosis (**Table 2B**).

**Table 2A:** Table 2A Results

Follow-up period (months)	49.2±21.5 (20–78)
Technical success (%)	12 (92.3)
Clinical success (%)	10 (76.9)
Complications	
Stroke	0
Paraplegia	0
Renal failure	0
Respiratory failure	0
Vascular injury	0
Graft infection	0
Rupture	0
Aortic-related mortality (%)	0
All-cause mortality (%)	4 (30.7)
Additional unplanned aortic surgery (%)	4 (30.7)

**Table 2B:** Table 2B Summary of additional unplanned aortic intervention

Reason	Intervention
d-SINE, n=1	TEVAR
Type 3b endoleak, n=1	TEVAR
Aneurysm dilation with incomplete CP n=2	DAR Repeat CP with plug-in coil embolization, n=1
RTAD, n=1	TAR

d-SINE: distal stent-graft-induced new entry tear; DAR: descending artery replacement; RTAD: retrograde Type A dissection; TAR: total arch replacement; CP: Candy-Plug; TEVAR: thoracic endovascular aortic repair

**Table 2C:** Table 2C Number of cases with variations in false lumen (FL) area in aortic segment after reentry closure

	<10%	±10%	>10%	Aortic remodeling
Bronchial bifurcation	6 (46.2)	4 (30.7)	3 (23.1)	7 (53.8)
Valsalva sinus	6 (46.2)	6 (46.2)	1 (7.6)	7 (53.8)
Celiac trunk	6 (46.2)	10 (76.9)	2 (15.5)	7 (53.8)
Infrarenal abdominal aorta	1 (7.6)	10 (76.9)	2 (15.5)	3 (23.1)

Ten patients (76.9%) achieved complete FL thrombosis above the diaphragm, while seven patients (53.8%) had aortic remodeling at 3 sites except for infrarenal abdominal aorta before and after reentry closure (**Table 2C**).

**Figure 2:**
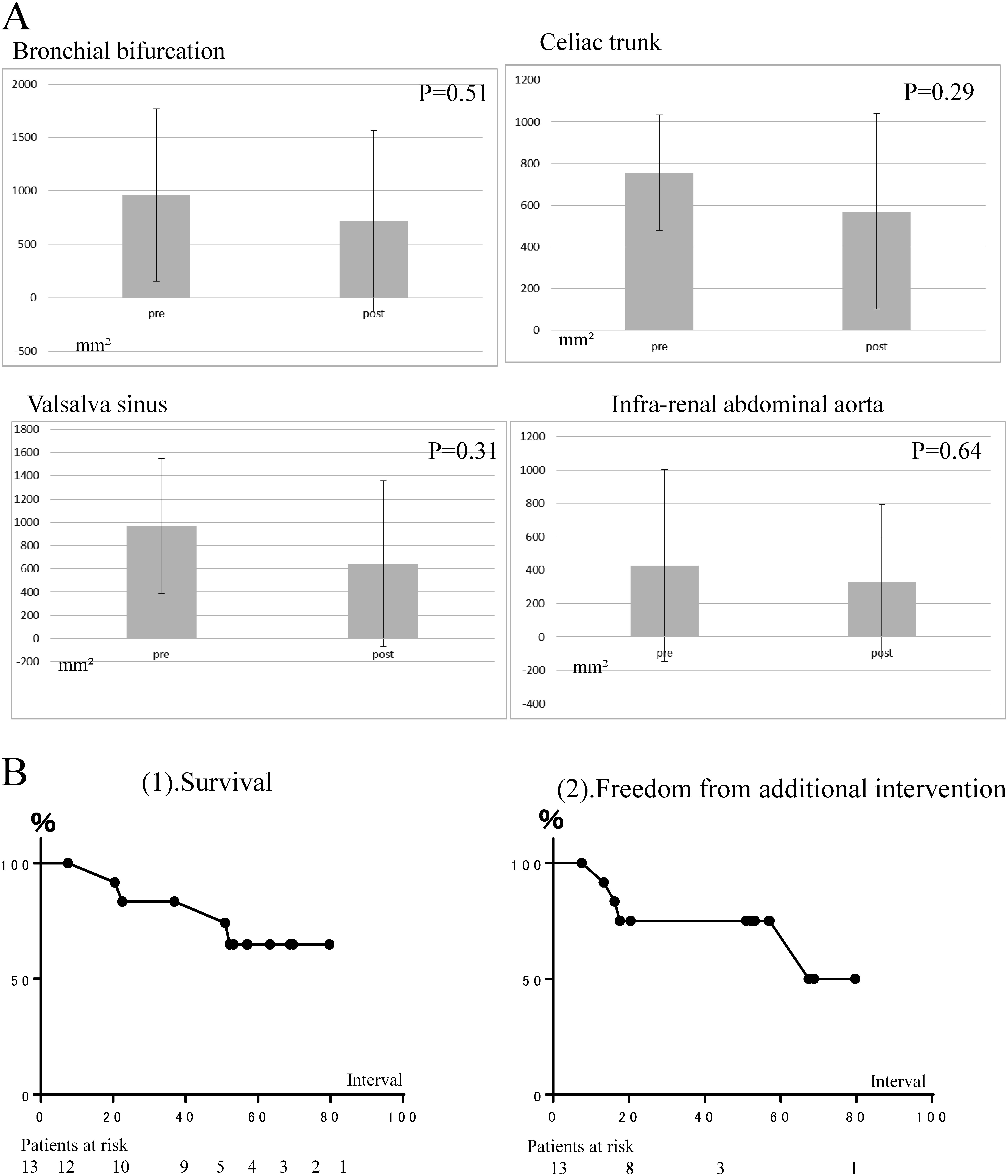
Fig. 2 (**A**) The change in mean false lumen (FL) area in the mean observation period of 49.2 months before and after the reentry closure. There was no statistically significant difference. (**B**) Kaplan–Meier analysis of survival (1) and freedom from additional intervention (2) after thoracic endovascular aortic repair with reentry closure.

**Figure 2A** shows the change in the mean FL area in all patients before and after reentry closure. The following FL mean volumes were recorded: preoperative 962.2±806 mm^3^, postoperative 719±845.2 mm^3^ (P=0.51) in bronchial bifurcation; preoperative 969.1±582.5 mm^3^, postoperative 642.4±712.7 mm^3^ (P=0.31) in Valsalva sinus; preoperative 756.9±277.5 mm^3^, postoperative 570.1±467.8 mm^3^ (P=0.29) in celiac trunk; and preoperative 427.7±577.6 mm^3^, postoperative 330.4±462.2 mm^3^ (P=0.63) in infrarenal abdominal aorta. The FL cross-sectional area was evaluated in contrast to CT before and after reentry closure, but the difference was not statistically significant.

Three patients showed an enlargement tendency because of endotension, which was detected by diagnostic imaging, despite achieving complete FL thrombosis. Two cases died during consideration for open surgery: one died due to myelodysplastic syndrome and the other died due to malignant lymphoma. Another one of the three is under currently follow-up. Three patients with incomplete FL thrombosis exhibited FL enlargement. Among these, one became an open conversion and descending aorta replacement was performed, and one died due to the progression of preexisting interstitial pneumonia. Meanwhile, the remaining case was under follow-up and was scheduled for thoracic abdominal replacement.

The 5-year overall survival rate was 64.8% (**Fig. 2B-1**). One patient died from acute interstitial pneumonia. No aortic-related deaths were observed during the study period. The 5-year freedom from additional interventions rate was 66.7% (**Fig. 2B-2**).

## Discussion

The main drawback of using TEVAR for CTBAD is the difficulty in controlling FL pressurization because of persistent retrograde FL flow to the intercostal arteries from multiple reentries, such as abdominal branches.^4)^ In other words, the closure of primary entry in the chronic phase alone cannot be expected to cause aortic remodeling.^19)^ One contributing factor is the existence of complex reentries, including remaining abdominal branches.^20)^ In our study, therapeutic interventions for the remaining reentries were required after an average of 40.7 months during the follow-up period even though the first primary entry was closed in seven cases.

Furthermore, the primary entry was covered by TEVAR based on preoperative diagnostic imaging to establish the treatment strategy. The reentry from the abdominal branched blood vessel and abdominal aorta to the iliac artery was handled by the procedure shown in **Fig. 1A** and **Table 1B**. The pressurization of FL above the diaphragm by retrograde flow through the reentry was controlled by the Candy-Plug method.^7)^

The optimal timing of TEVAR for only primary entry or in combination with reentry closure remains under debate. In our cases, we performed TEVAR and reentry closure as two-staged operations in 7 out of 13 patients. Treatment strategies require reconsideration since new reentries from intercostal and lumbar arteries that were not closed before the initial TEVAR may exist. In addition, spinal cord ischemia was considered.^21)^

Static obstruction is a problem of reentry closure if the abdominal branch spans the FL alone or both the true lumen and the FL. The bare metal stent was placed in the true lumen and coiling for FL throughout the space of the bare stent was performed in the case of the celiac artery. It seems that aortic remodeling is good even for branched abdominal vessels by placing a covered stent in the acute phase,^13)^ but we believed that in the chronic phase obtaining aortic remodeling would be difficult because the flap becomes hard, even in the abdominal branch. Therefore, we devised this procedure. The celiac trunk is obstructed by the stent-graft when the celiac artery is the complete perfusion of the FL, confirming the anatomical traffic between the celiac trunk and the superior mesenteric artery. Then, coil embolization for the origin of the celiac trunk was performed.^15)^ In cases of the renal arteries, a bare stent was only placed in the true lumen if it was narrowed by a static lesion in preoperative CT (**Fig. 1A-d**). Bel et al. have reported a spot stenting method.^13)^ However, our country does not have insured devices. In addition, the long-term prognosis of the part of the stent that protrudes into the aorta during stent placement in the branched vessel is unknown.^13,16)^ In our cases, no problems were detected in the observation period.

The FL flow through the intercostal artery near the abdominal branch was closed by several methods. The Excluder aortic cuff was used for closure when it was detained on the true lumen site. The abdominal branched blood flow was protected by the chimney method when there was a concern that other branch arteries would be closed.^17)^ If there was a concern regarding the occlusion of branched blood vessels because of this procedure, Excluder aortic cuff was detained on the FL side to close the origin of the intercostal artery (**Figs. 1A-g** and **1A-h**). Candy-Plug was detained on the FL side. The Petticoat technique was also used to maintain the vessel diameter of the true lumen on the same side.^22)^

In practice, the infrarenal proximal extension device (Endologix AFX Unibody Endograft system, Irvine, CA, USA) was removed from the sheath once, leaving the anastomosis portion of polytetrafluoroethylene graft 5 mm from the proximal and distal side. It was then mounted again in the sheath and extended across the abdominal branch (celiac trunk, superior mesenteric artery, and bilateral renal artery; **Figs. 1A-b, 1A-h,** and **1A-i**).

The Endurant aortic extension (Medtronic Vascular, Santa Rosa, CA, USA), whose length is easily adjusted, was used for reentry closure from below the renal artery to the terminal abdominal aorta or the bilateral common iliac artery.^16,17)^ The Endurant aortic extension was detained to close the plucked lumber artery and inferior mesenteric artery. The size was selected to be 90% lower or of the same size as the long axis of the true lumen site.

In the case of chronic aortic dissection, the true lumen of the abdominal artery to the terminal aorta site is often narrowed. In one case in which reentry was recognized from the common iliac to the external iliac artery, we performed the embolization of the internal iliac artery and reentry closure using the Double D technique.^10)^ Both methods are thought to have contributed to FL thrombosis without endoleak.

The definition of aortic remodeling after TEVAR for chronic aortic dissection has not been disclosed.^23)^ Mani et al. suggested that aortic remodeling is an indicator of the survival rate of midterm results. The maximum diameter of the descending aorta was reduced by more than 5 mm in the last postoperative image compared with the preoperative image.^5)^ However, the true lumen expands with aortic remodeling. Hence, the FL shrinks relatively, and it is difficult to say that the aortic remodeling is achieved only by considering the aortic diameter. In their study, Melissano et al. calculated the volume of the true and FL cavity and showed that a 10% reduction was obtained after the operation.^9)^ Yang et al. measured the maximum diameter of the true and FL at four locations (the left subclavian and pulmonary artery, diaphragm, and celiac trunk) and showed significant changes of 5 mm or more.^24)^

We focused on the FL cross-sectional area of the bronchial bifurcation, Valsalva sinus, above the diaphragm, the celiac trunk, and infrarenal abdominal aorta level under the diaphragm. Aortic remodeling was confirmed if the FL cross-sectional area was reduced by 5% or even slightly in the final image in comparison with the preoperative image or if it was invariant and complete thrombosis was obtained (**Table 2C**).^9)^

However, retrograde aortic dissection on the proximal side of the detention site of the stent-graft and new entry tear on the distal side, which can occur after TEVAR, are serious problems.

The absence of spinal cord ischemia and respiratory complication is a benefit of endovascular treatment even if the patient background is considered, given the early results of this study. In the midterm results of reentry closure, 10 patients (76.9%) had complete FL thrombosis via various endovascular treatment methods. No aortic-complication-related deaths, such as aortic rupture, were observed for an average of 49.2 months. In addition, the 5-year freedom from additional intervention rate of 66.7% can be considered good. Thus, an improvement in the devices used to treat FL occlusion is desirable. All cases with enlarged FL cross-sectional areas had insufficient flow control from reentry at the Candy-Plug detained site. In the future, we believe that the control of FL blood flow will be better if other commercial devices can have a wider contact area with the FL vessel wall. We are looking forward to the development of other devices and their clinical trials.

The limitations of this study are as follows: First, this was a retrospective analysis with a small sample. Second, a selection bias regarding the preoperative diagnostic imaging and the patients’ respiratory dysfunction was present. Lastly, the observation period was short and verification of the results by conducting a study with a longer follow-up period is required.

## Conclusions

We reported the midterm results of TEVAR for CTBAD with aneurysmal dilation. Various endovascular reentry closure techniques suggest preventing FL dilatation or rupture, but the revision of our devices and further research with more patients and a longer follow-up period are required.

## Additional Remarks

The abstract of this paper was presented at the 74th Annual Meeting of the Japanese Society of Thoracic Surgery in Tokyo in October 2021.
